# Remobilization of *Tol2 *transposons in *Xenopus tropicalis*

**DOI:** 10.1186/1471-213X-10-11

**Published:** 2010-01-22

**Authors:** Donald A Yergeau, Clair M Kelley, Emin Kuliyev, Haiqing Zhu, Amy K Sater, Dan E Wells, Paul E Mead

**Affiliations:** 1 Department of Pathology, St. Jude Children's Research Hospital, 262 Danny Thomas Place, Memphis, TN 38105, USA; 2 Department of Biology and Biochemistry, University of Houston, Houston, TX 77204, USA

## Abstract

**Background:**

The Class II DNA transposons are mobile genetic elements that move DNA sequence from one position in the genome to another. We have previously demonstrated that the naturally occurring *Tol2 *element from *Oryzias latipes *efficiently integrates its corresponding non-autonomous transposable element into the genome of the diploid frog, *Xenopus tropicalis. Tol2 *transposons are stable in the frog genome and are transmitted to the offspring at the expected Mendelian frequency.

**Results:**

To test whether *Tol2 *transposons integrated in the *Xenopus tropicalis *genome are substrates for remobilization, we injected *in vitro *transcribed *Tol2 *mRNA into one-cell embryos harbouring a single copy of a *Tol2 *transposon. Integration site analysis of injected embryos from two founder lines showed at least one somatic remobilization event per embryo. We also demonstrate that the remobilized transposons are transmitted through the germline and re-integration can result in the generation of novel GFP expression patterns in the developing tadpole. Although the parental line contained a single *Tol2 *transposon, the resulting remobilized tadpoles frequently inherit multiple copies of the transposon. This is likely to be due to the *Tol2 *transposase acting in discrete blastomeres of the developing injected embryo during the cell cycle after DNA synthesis but prior to mitosis.

**Conclusions:**

In this study, we demonstrate that single copy *Tol2 *transposons integrated into the *Xenopus tropicalis *genome are effective substrates for excision and random re-integration and that the remobilized transposons are transmitted through the germline. This is an important step in the development of 'transposon hopping' strategies for insertional mutagenesis, gene trap and enhancer trap screens in this highly tractable developmental model organism.

## Background

Transposons are naturally occurring mobile genetic elements and have been used as tools to experimentally modify the genomes of a wide range of model organisms. Used extensively in insects and plants for decades, recent advances in transposon-based technologies have expanded their use to vertebrate systems. DNA-based 'cut-and-paste' transposon systems have been adapted to provide efficient transgenesis tools that can stably integrate an exogenous cargo into the genome without incorporation of plasmid vector sequences. *Sleeping Beauty (SB)*, a member of the *Tc1*/*mariner *family of transposable elements, was molecularly reconstructed from an ancient inactive element found in Salmonoid fishes [1]. *SB *has been used extensively in the mouse for cancer gene discovery [2-5], in *Xenopus *for transgenesis [6-8], and zebrafish for gene and enhancer trap screens [9-13]. The *piggyBac *transposon system from the cabbage looper moth [14] has been shown to efficiently insert into vertebrate [15] and invertebrate genomes [16-20]. Most recently, *piggyBac *was used to non-virally integrate key developmental genes to reprogram induced pluripotent stem (iPS) cell lines [21,22].

Isolated from the teleost medaka (*Oryzias latipes*) as the first functional vertebrate autonomous transposase [23], *Tol2 *is a member of the *hAT *family (*hobo *from *Drosophila*, *Ac *from maize, and *Tam3 *from snapdragon) of transposable elements. Genetic manipulation of the *Tol2 *system has produced a non-autonomous element suitable for transgenesis applications. The non-autonomous *Tol2 *element requires active *Tol2 *transposase to be supplied *in trans *for integration of cargo DNA flanked by the *Tol2 *transposon terminal end repeats into the genome [24-26]. *Tol2 *acts as a 'cut-and-paste' transposase integrating its transposable element randomly throughout the genome and creates a characteristic eight (8) base pair target site duplication (TSD) of host genomic DNA flanking the integration site. *Tol2 *has been successfully used for integration of exogenous DNA into zebrafish for transgenesis [27,28] and enhancer and gene trap strategies [29-32]. Previously, we demonstrated that *Tol2 *effectively integrates a GFP reporter construct driven by the ubiquitous EF-1α promoter (*Tol2*XIG) into the amphibian model organism *Xenopus tropicalis *[33]. The *Tol2 *transposable element is stable in the *Xenopus tropicalis *genome and is transmitted to progeny at expected Mendelian ratios following the F_1 _generation.

Once integrated into the genome, *Tol2 *non-autonomous transposons can act as substrates for remobilization only in the presence of active *Tol2 *transposase [34]. Methods to re-express the *Tol2 *transposase in vertebrates include injection of the *Tol2 *transposase mRNA directly into one-cell embryos carrying a *Tol2 *transposon and by the development of *Tol2 *transposase-expressing transgenic lines that can be outcrossed with *Tol2 *transposon substrate lines. The transposase injection-based method is time-consuming due to the injection of the transposase mRNA directly into individual embryos whereas the breeding-based double-transgenic strategy is potentially more efficient for large-scale remobilization screens. In the zebrafish, resident *Tol2 *transposons can be remobilized to novel loci in the presence of the *Tol2 *transposase [29,35]. Both injection and *in vivo *expression of the *Tol2 *transposase in *Tol2 *transposon-haboring animals resulted in novel re-integration events throughout the zebrafish genome [35]. Remobilized *Tol2 *transposons in the zebrafish can result in novel tissue-restricted GFP expression profiles that can be used for the study of organogenesis [29,36-38]. A recent report from the Largaespada laboratory also demonstrates that *Tol2 *transposons can be remobilized in the germline of mice [39].

To determine whether re-expression of the *Tol2 *transposase can remobilize a transposon present in the *Xenopus tropicalis *genome, we injected *Tol2 *transposase mRNA into one-cell embryos harvested from transgenic frogs heterozygous for a single *Tol2 *transposon and analyzed the developing tadpoles for remobilization. We identified at least one novel integration site in each embryo injected with the *Tol2 *transposase mRNA. This confirmed that *Tol2 *transposons resident in the frog genome are effective substrates for remobilization strategies. To test whether these remobilization events can be transmitted to progeny, we raised *Tol2 *transposase injected tadpoles to adulthood and outcrossed the 'remobilized frogs' to wild type animals. Progeny from one animal, 12M2♀5, exhibited a GFP expression pattern not seen in the *Tol2 *12M2 transposon line used for injection. Analysis of progeny from 12M2♀5 showed germ-line transmission of four novel integration events and that these four integration sites can be independently segregated by outcross. Several of these novel integration sites are located within genes suggesting that *Tol2 *can potentially target genes for insertional mutagenesis strategies. In addition, we show that the parental *Tol2 *integration site is frequently maintained in remobilized progeny suggesting that *Tol2*, like other *hAT *family members [40-42], catalyzes transposition during cell division. Overall, our study provides the first evidence that *Tol2 *can remobilize its cognate transposon to novel loci in the genome of *Xenopus tropicalis *and thus provides a powerful genetic tool to manipulate the frog genome for gene and enhancer trapping and insertional mutagenesis.

## Results

### Integration of *Tol2*XIG transposons at discrete loci result in different GFP expression patterns

We previously reported the efficient integration and germline transmission of a *Tol2 *GFP reporter transposon construct into the genome of the frog *Xenopus tropicalis *[33]. In our previous studies, we showed that *Tol2 *transposons are stable in the genome and are transmitted at Mendelian ratios following the F_1 _generation. Preliminary analysis of line 12M had indicated that this founder contained independently-segregating *Tol2*XIG transposon alleles [33]. Sequential outcross of the 12M line resulted in segregation of these alleles to reveal four distinct GFP expression patterns corresponding to four unique integration events in the 12M founder (see [Additional file 1: Supplemental data] and [Additional file 2: Supplemental fig. S1] and [Additional file 3: Supplemental fig. S2] for details).

### Injection-mediated *Tol2* transposon remobilization in *Xenopus tropicalis*

**Figure 1:**
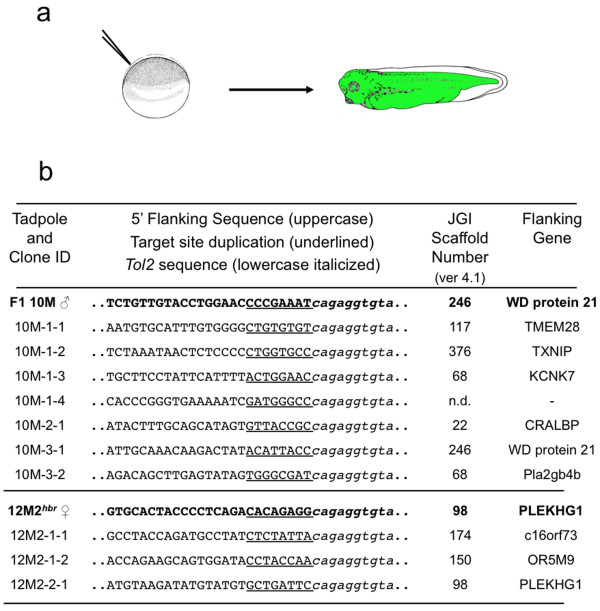
**Somatic remobilization of a single copy *Tol2 *transposon in *Xenopus tropicalis***. (a) Schematic of the micro-injection remobilization strategy. Embryos were injected with synthetic *Tol2 *transposase mRNA at the one-cell stage and allowed to develop to swimming tadpole stage before genomic DNA was harvested for analysis of the integration sites by EPTS LM-PCR. (b) Sequence of the *Tol2 *integration sites identified in the 'remobilized' tadpoles. Two independent single transposon integration sites (10M and 12M) were used to demonstrate somatic remobilization of *Tol2 *transposons in *Xenopus tropicalis*. The parental integration sites are shown in bold. Examples of novel re-integration sites, indicating remobilization of the parental transposon, are listed below the parental site and are labelled to identify the tadpole and clone number (for example, 10M-tadpole number-clone number). Multiple remobilization events could be identified in individual tadpoles, tadpole 10M-1 had four novel integration events, suggesting that multiple independent remobilizations had occurred in discrete blastomeres during early development. All tadpoles analyzed (18/18 = 100%) had at least one novel re-integration event. The parental transposon could be remobilized from either male or female *Tol2*XIG F_1 _transgenic donor animals. The genomic sequence flanking the 5'-end of the transposon is shown in capital letters and the eight base pair target site duplication (TSD) is underlined. The transposon sequence in depicted by lower case italics text. The transposon flanking sequences were compared to the *Xenopus tropicalis *genome sequence (JGI assembly v4.1) to identify the scaffold identity of the integration site and to identify flanking genes; the gene nearest to the transposition site is listed in the table.

**Figure 2:**
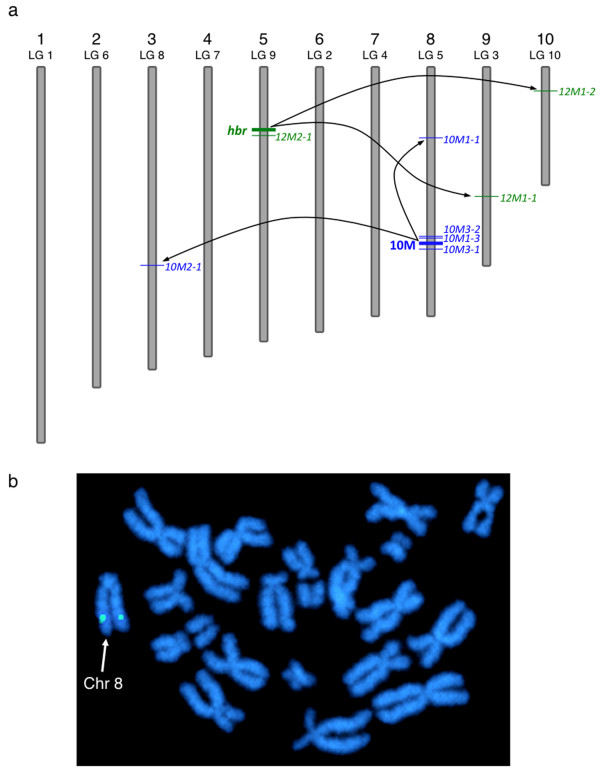
**Chromosomal location of *Tol2 *transposon insertions in the *Xenopus tropicalis *genome**. (a) Schematic representation of *Xenopus tropicalis *chromosomes and the relative location of the *Tol2 *integration sites (not to scale). Chromosomal assignments were predicted using the scaffold identity revealed by comparison of the transposon flanking sequence with the *Xenopus tropicalis *genome sequence (JGI v4.1 assembly). The position of the *Tol2 *integration on each chromosome was estimated from the map position of the corresponding scaffold. The parental integration sites (10M and 12M (*hbr*)) used for somatic remobilization are depicted by the thick lines and are labeled on the left side of the chromosome. Examples of remobilized somatic transposition events listed in Figure 1b are labelled on the right hand side of the chromosome. Mapping the integration sites provided evidence for both 'local hopping' and random re-integration throughout the genome. (b) Fluorescent in situ hybridization (FISH) analysis of the 10M line confirms the location of the *Tol2*XIG transposon on the long arm of chromosome 8 (linkage group 5). Metaphase spreads prepared from heterozygous 10M tadpoles were hybridized with a digoxigenin-labeled GFP probe and counterstained with DAPI.

To test the ability of a resident *Tol2 *transposon to be remobilized *in vivo*, we performed a pilot injection-based screen using F_2 _progeny derived from two founder lines, 10M and 12M [33]. To simplify the subsequent analysis, the F_1 _transgenic parents used for this experiment each harboured a single transposon integration event. *Xenopus tropicalis *embryos at the one-cell stage were injected with 500 pg of *in vitro *transcribed *Tol2 *transposase mRNA and genomic DNA was harvested from individual tadpoles at stage 40 (Figure 1a). EPTS LM-PCR was performed to identify the genomic sequence flanking the 5' end of the *Tol2 *transposon arm. The results of somatic remobilization of the *Tol2 *transposon in two different founder lines, 10M and 12M2 (*hbr*) are shown in Figure 1b. Eighteen individual stage 40 tadpoles were analyzed by EPTS LM-PCR [43] and 100% (18/18) of the embryos had at least one new integration site and several tadpoles had multiple, novel integration events. For example, in embryo 10M-1, four integration sites were identified that were different from the parental scaffold 246 integration site and each new integration had a unique 8 base pair (bp) target site duplication (TSD). In addition, re-integration of the *Tol2 *transposon was observed in individual embryos on the same scaffold as the parental integration event. Genomic DNA prepared from embryos 10M-3 and 12M-2 harboured remobilization events of the *Tol2 *transposon on the same scaffold as the parental locus (the 10M line has an integration on scaffold 246 that maps to the long arm of chromosome 8 (Figure 2b); and the 12M F_1 _female frog (12M2) used in this experiment harbours an integration on scaffold 98 (the *hbr *allele)). This indicates that *Tol2*, like other DNA 'cut-and-paste' transposases, can catalyze remobilization events that re-integrate near the site of excision, a phenomenon referred to as 'local hopping'. The scaffold identity, revealed by BLASTN search of the flanking sequence with the *Xenopus tropicalis *genome assembly v4.1, was "mapped" to the chromosomal assignment by comparing the scaffold number with the list of scaffolds mapped to unique linkage groups on the *Xenopus tropicalis *genetic map (Figure 2). We have adopted the modified *Xenopus tropicalis *chromosome nomenclature described by Khokha and colleagues [44]. This analysis indicated that, in addition to 'local hopping', re-integration events had occurred throughout the genome. Thus, we can conclude that expression of the *Tol2 *transposase by microinjection can remobilize a resident *Tol2 *transposon in an established founder line. Furthermore, we were able to remobilize the *Tol2 *transposon substrate in embryos harvested from either male or female heterozygous *Tol2*XIG transgenic parents.

### Analysis of transposase injected tadpoles for germline transmission of remobilized *Tol2* transposons

In a pilot injection-based remobilization experiment we used an F_1 _female, 12M2, that inherited the *hbr *integration site alone, confirmed by multiple outcrosses, genomic PCR, Southern blot and GFP visualization ([Additional file 2: Supplemental Fig. S1a] and data not shown). Injected embryos from female 12M2 were divided into two pools, one group was harvested for somatic remobilization studies (Figure 1b) and the remaining pool was allowed to develop to adult stage. The adult 'remobilized' 12M2 F_2 _frogs were outcrossed to wild type animals to determine whether the remobilized transposons are passed through the germline.

Seven GFP-positive F_2 _'remobilized' adults were successfully out-crossed to wild type frogs to test for germline transmission of remobilized *Tol2 *transposons (table 1). Due to the mosaic nature of transgenic founder (P_0_) animals derived from the co-injection of transposon plasmid substrate and synthetic *Tol2 *mRNA, we anticipated that remobilization by injecting *Tol2*XIG transgenic embryos with *Tol2 *transposase mRNA would result in mosaic animals. This is due to the ability of *Tol2 *to catalyze remobilization events in discrete blastomeres during the early stages of development, and thus the resulting animal, and its germline, is likely to be mosaic for the parental and novel *Tol2*XIG transposon integration events. To examine this, we analyzed tissues from an adult remobilized animal (12M2♀3) that had died following outcross. Integration site analysis was performed on genomic DNA prepared from multiple tissues to verify the mosaic nature of the injection-mediated remobilization products. Novel re-integration events were identified in discrete somatic tissues of this animal. EPTS LM-PCR confirmed at least two remobilization events in genomic DNA isolated from the right lung (scaffold 2:5750172, chromosome 9/LG 3 in the first intron of saccharopine dehydrogenase and in scaffold 486:789402, into a splice isoform of a GTPase activating Rap/Ran-GAP domain-like 1 protein). Interestingly, the integration site into scaffold 486 is on chromosome 5/LG 9, the same chromosome as the *hbr *parental integration site. Analysis of progeny obtained from animal 12M2♀3 show no germline remobilization (data not shown). Likewise, integration site analysis of genomic DNA harvested from the left and right ovaries indicated no evidence for remobilization in the germline (data not shown). This result confirms remobilization of the resident transposon in somatic tissues, consistent with our earlier studies (see Figure 1b), but that remobilization events occurred in a blastomere leading to formation of the right lung and not in the germline.

**Table 1:** Analysis of outcrossed remobilization lines in *Xenopus tropicalis*

*Remobilized Frog*	*GFP +*	*GFP -*	*Total*	**%*GFP+ ***	**%*GFP - ***	_χ_^*2 *^*(df = 1)*
12M2♂1	1477	1536	3013	49.02	50.98	1.15

12M2♀2	379	374	753	50.33	49.67	0.03

12M2♀5*	**4361**	**3543**	**7904**	**55.17**	**44.83**	**84.7**

12M2♀6	480	605	1085	44.24	55.76	14.4

12M2♀14	323	325	648	49.85	50.15	0.01

12M2♂16	2365	2119	4484	52.74	47.26	13.5

12M2♂17	1020	936	1956	52.15	47.85	3.61

* Four novel remobilization events have been identified in progeny from 12M2♀5 (bold).

Table 1 shows the rates of transgenesis from outcrosses of seven adult 'remobilized' frogs. As the parental frog used to generate embryos for this experiment contained a single copy of the *Tol2XIG *transposon, we expected 50% of the progeny to have the dominant GFP allele. The data presented in table 1 shows that the observed frequency of GFP-positive tadpoles in the outcross population frequently varied from the expected 1:1 ratio of GFP-positive and GFP-negative animals. To date, we have cloned novel integration events from one of these 'remobilized' animals, 12M2♀5 (table 1, bold).

### Characterization of F_1 _progeny from remobilized founder 12M2♀5

**Figure 3:**
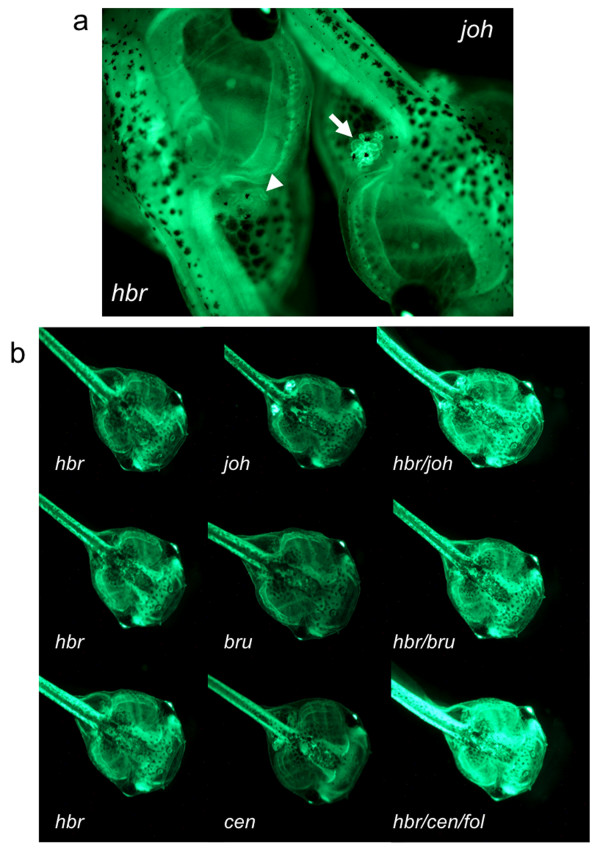
**Remobilized *Tol2 *transposons are transmitted through the germline and result in novel GFP expression patterns**. Fluorescent photomicrographs of sibling GFP-positive tadpoles from the outcross of remobilized frog 12M2♀5. (a) The parental integration pattern (*hbr*) is shown in the tadpole on the left (anterior facing upper left) and the novel GFP expression pattern is shown on the right (*jovan heat*, *joh*; anterior facing lower right). The tadpoles were oriented to juxtapose the kidneys for comparison of the different levels of GFP expression in the kidneys of the two lines. Note the intense GFP expression in the kidney of the *joh *tadpole (white arrow) compared to the kidney of the *hbr *tadpole (white arrowhead). (b) Representative GFP expression patterns from the outcross of 12M2♀5. Left column shows tadpoles (stage 48) with the parental (*hbr*) GFP expression pattern. Four novel transposon insertion sites were identified in the progeny of 12M2♀5 and were named *jovan heat *(*joh*), *brut *(*bru*), *centaure *(*cen*) and *follique *(*fol*). Each of the novel integration events has a subtle difference in the resulting GFP expression pattern. All tadpoles are oriented with the anterior facing towards the lower right corner of each panel.

Visual examination of all offspring derived from the outcross of the 'remobilized' 12M2 adults showed that most of the progeny have the same expression pattern of GFP as the *hbr *founder (Additional file 2; Supplemental fig. S1a]. One adult animal, 12M2♀5, produced progeny with a GFP expression pattern different from its parent. Approximately 4.7% (39 of 828) of the progeny from the initial outcross of 12M2♀5 had GFP expression visible in the kidneys (Figure 3a, white arrow) compared to GFP positive siblings without expression in the kidney (Figure 3a white arrow head). Approximately 49% (406 of 828) of the 12M2♀5 outcross was GFP-positive but lacked reporter expression in the kidney. GFP expression in the kidney was not observed in other lines derived from founder 12M (*hbr*, *slp, grb *and *chs*). Outcross of the 'donor' female 12M2 with a wild type *Xenopus tropicalis *male produced embryos containing only the *hbr *locus with no GFP expression within the kidney (Figure 3a, left embryo). The visual examination was confirmed by PCR and Southern analyses and showed that the 12M2 frog contained only the *hbr *allele. We therefore concluded that the 'kidney-positive' population represented progeny containing a novel remobilization event.

**Figure 4:**
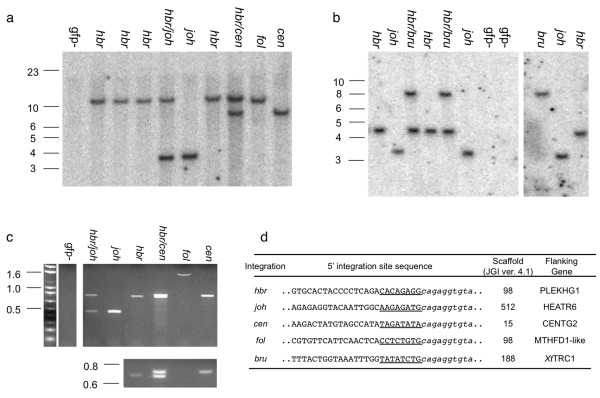
**Molecular analysis of the progeny from 'remobilized' frog 12M2♀5**. Southern blot analysis was performed on genomic DNA harvested from individual tadpoles and digested with either *Bam*HI (a) or *Bgl*II (b) and probed with a radiolabeled GFP probe to demonstrate the inheritance of novel transposon integration events. EPTS LM-PCR was used to amplify the genomic sequences flanking the 5' end of the *Tol2 *transposon. The amplified bands were resolved on agarose gels (c) and subcloned prior to sequence analysis. Sequence analysis (d) revealed that the novel remobilization events were the result of transposition as the insertion sites were flanked by the expected 8 bp target site duplications (TSD, underlined). The flanking genomic sequence is depicted by the capitalized text and the *Tol2 *sequence is shown in lowercase italics. The *fol *integration event was mapped to the same scaffold as the parental (*hbr*) integration site and represents a 'local hopping' transposition.

Additional outcross of *Xenopus tropicalis *12M2♀5 produced tadpoles with different GFP expression levels (Figure 3b). Southern blot analysis (Figure 4a and 4b) was performed on genomic DNA prepared from individual tadpoles and indicated the presence of novel bands due to remobilization events. We identified numerous embryos with GFP-positive Southern blot banding patterns that were different from the parental *hbr *locus (*Bam*HI digest for *hbr *= ~10.5 kb and *Bgl*II digest for *hbr *~4.5 kb). EPTS LM-PCR (Figure 4c) was performed on individual embryos exhibiting unique Southern blot profiles. PCR amplification of sequences flanking the *Tol*2XIG transposon in the tadpoles harbouring novel insertion sites confirmed that remobilization had occurred (for example, compare the ETPS LM-PCR product in the *hbr *lane with the *joh *lane, Figure 4c). The EPTS LM-PCR products were cloned and sequence analysis revealed novel integration sites that differ from the parental *hbr *allele (Figure 4d). Genomic PCR using primers designed to the corresponding scaffold sequence outside the cloned EPTS LM-PCR product confirmed the integration site and was further verified by PCR cloning the transposon-genomic flanking sequence on the other side of the *Tol2*XIG transgene. We have identified four independently-segregating remobilization events from 12M2♀5 and named them *jovan heat *(*joh*, scaffold 512), *centaure *(*cen*, scaffold 15/chromosome 9), *brut *(*bru*, scaffold 188/chromosome 4), and *follique *(*fol*, scaffold 98/chromosome 5). Examples of the GFP expression pattern for each of these new integration sites in developing tadpoles is depicted in Figure 3b.

Integration site analysis determined that embryos that have high-levels of GFP in the pronephric kidney contained a transposon that had integrated into the genomic locus of a putative gene encoding a novel HEAT motif-containing protein (scaffold 512:565147). The HEAT motif forms repetitive helical structures common to **H**untington protein, **E**longation factor 3, PP2A (**A **subunit of protein phosphatase 2A) and **T**or (target of rapamycin) [45], so we have named this *Tol2 *remobilization event *jovan heat *(*joh*). The integration site is 1032 base pairs downstream of exon 9 and 77 bp upstream of exon 10 of this predicted thirteen exon gene. The *joh *integration event occurred in the 3'-end of a predicted HEAT motif-containing protein, however, *in situ *hybridization [46] with antisense RNA probes for this gene did not exhibit robust expression in the developing kidney (data not shown). The nearest flanking gene downstream of the *joh *integration site is HNF1β, a gene that is highly expressed in the developing *Xenopus *kidney ([47] and [Additional file 4: Supplemental fig. S3]) and is a likely candidate for the gene driving the GFP expression pattern observed in *joh*. This data indicates that the gene closest to the transposon integration site may not be responsible for the observed GFP expression pattern as the minimal EF-1α promoter in the *Tol2*XIG transposon may be influenced by regulatory elements of more distal flanking genes.

**Figure 5:**
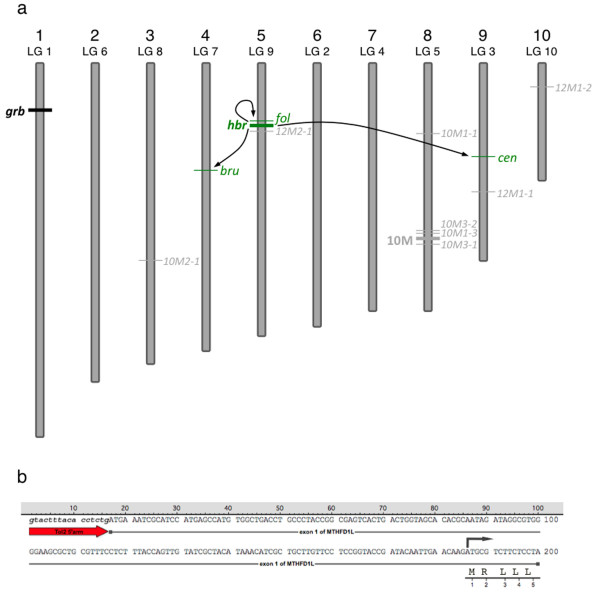
**Chromosome map of *Tol2 *transposon insertions transmitted through the germline**. (a) Schematic representation of *Xenopus tropicalis *chromosomes and the relative location of the *Tol2 *integration sites (not to scale). Chromosomal assignments were predicted using the scaffold identity revealed by comparison of the transposon flanking sequence with the *Xenopus tropicalis *genome sequence (JGI v4.1 assembly). The position of the *Tol2 *integration on each chromosome was estimated from the map position of the corresponding scaffold. The parental integration site, *hbr *used for the germline remobilization experiment, is depicted by the thick green line and is labeled on the left side of the chromosome. Examples of remobilized transposition events that are passed through the germline are in green and are labeled on the right hand side of the corresponding chromosome. Mapping the integration sites provided evidence for both 'local hopping' and random re-integration throughout the genome. Remobilization events are indicated by the arrows. (b) Sequence analysis of the EPTS LM-PCR product from the *fol *allele reveals integration of the *Tol2*XIG transposon at the beginning of the first exon of the MTHFD1L gene. The sequence of the 5'-end of *Tol2*XIG is in lowercase italics and represented by the red arrow. The integration event has occurred at the first nucleotide of exon 1 (uppercase text) of the MTHFD1L gene, 169 bp upstream of the translation start codon, depicted by the dark arrow above the sequence. The first five amino acids of the MTHFD1L gene are shown in single letter code below the corresponding codons.

The second remobilization event, *follique *(*fol*), occurred in scaffold 98:2697817, approximately 153 kb away from the parental *hbr *locus and represents a 'local hopping' event. Here, *Tol2 *has integrated immediately adjacent to the first exon (169 base pairs upstream of the translational start site (Figure 5b)) of the predicted *Xenopus tropicalis methylenetetrahydrofolate dehydrogenase (NADP + dependent) 1-like *(MTHFD1L) gene. The MTHFD1L enzyme is important in folate metabolism and is required for purine synthesis during embryogenesis and rapid cell growth [48]. Polymorphisms of MTHFD1L in humans are associated with disease susceptibility and embryonic abnormalities including neural tube defects [49]. The proximity of the *Tol2 *integration site to the transcription start site of this mRNA suggests that this integration may be mutagenic; a hypothesis that will be tested in the future by incross of heterozygous animals bearing single *Tol2*XIG *fol *alleles.

The *centaure *(*cen*) allele results from a *Tol2 *remobilization into scaffold 15:1853930 within a 10 kb intron of the predicted centaurin-γ-2 gene locus that encodes a large multi-domain ArfGAP membrane protein that contains ankyrin repeats and a pleckstrin homology domain [50].

EPTS LM-PCR clones of the *brut *(*bru*) allele contain repetitive sequences that align with repeat elements in scaffold 188. Here, the *Tol2*XIG re-integration site is within the genomic locus of a predicted gene encoding a member of the TRCI (**T**ransposon **R**elated to **C**ACTA **I**) family of transposases [51]. Analysis of the predicted amino acid sequence indicates that most of the putative *Xt*TRCI protein shares a high degree of homology to TRCs in zebrafish, sea urchin, *Drosophila*, mosquito, hydra and fluke, and is completely conserved in the transposase domains.

To determine whether excision of the *Tol2*XIG transposon from the parental locus resulted in a footprint, that is, the modification of the endogenous locus, we performed genomic PCR of the endogenous locus with primers that flank the *hbr *integration site on scaffold 98. The sequence of the PCR products from multiple tadpoles was identical to the wild type locus indicating that the excision reaction resulted in the precise removal of the *Tol2 *transposon with no apparent modification of the locus (genomic DNA from six GFP-positive tadpoles, with individual or combinations of the *fol*, *cen *and *joh *alleles, were PCR amplified and sequenced; data not shown). We also examined genomic DNA samples from the somatic remobilization experiments and were unable to detect modifications caused by excision of the *Tol2 *element (genomic DNA from eight 10M remobilized tadpoles was analyzed; data not shown).

To determine the percentage of the progeny from 12M2♀5 that inherit either the parental (*hbr*) allele or the four novel remobilized alleles (*joh*, *cen*, *fol *and *bru*) we sorted embryos into GFP-positive and GFP-negative populations and then randomly selected 126 GFP-positive tadpoles for analysis. Each tadpole was photographed and genomic DNA was prepared for molecular analyses. The genotype of each animal was determined by Southern blot and verified using genomic PCR. The data from this random sampling is shown in table 2. Approximately 78% (98 of 126) of the GFP-positive population from outcross of 12M2♀5 harbour the parental *hbr *allele alone. The remaining 22% (28 of 126) contained combinations of individual integration events (11 of 126 were *joh *only) or combination of two, or more, integrations (for example, *hbr*/*cen*/*fol *was found in 3 embryos out of 126). In this randomly selected sample of 126 embryos we failed to identify tadpoles containing only the *fol *integration site, however, this individual allele was found in other outcrosses of 12M2♀5 (see Southern blot, Figure 4a, *fol *lane). This data suggests that 22% of GFP-positive embryos from 12M2♀5 contain at least one novel remobilization event.

**Table 2:** Unbiased analysis of 126 GFP-positive tadpoles to determine the percentage of *Tol2 *transposons remobilized in 12M2♀5 progeny

	*Integration Site*	*Number of Progeny*	*Frequency*
Parental	*hbr *only	98/126	77.8

*Remobilization*	All remobilized	28/126	22.2

	*joh *only	11/126	8.7

	*cen *only	2/126	1.6

	*bru *only	3/126	2.4

	*fol *only	0/126	<1%*

	*hbr *+ *joh*	4/126	3.2

	*hbr *+ *bru*	5/126	4.0

	*hbr *+ *cen *+ *fol*	3/126	2.4

* Embryos containing the *fol *integration site in isolation were observed in additional outcrosses.

### Maintenance of the *joh* integration site in the genome of F_4 _*Xenopus tropicalis* embryos

**Figure 6:**
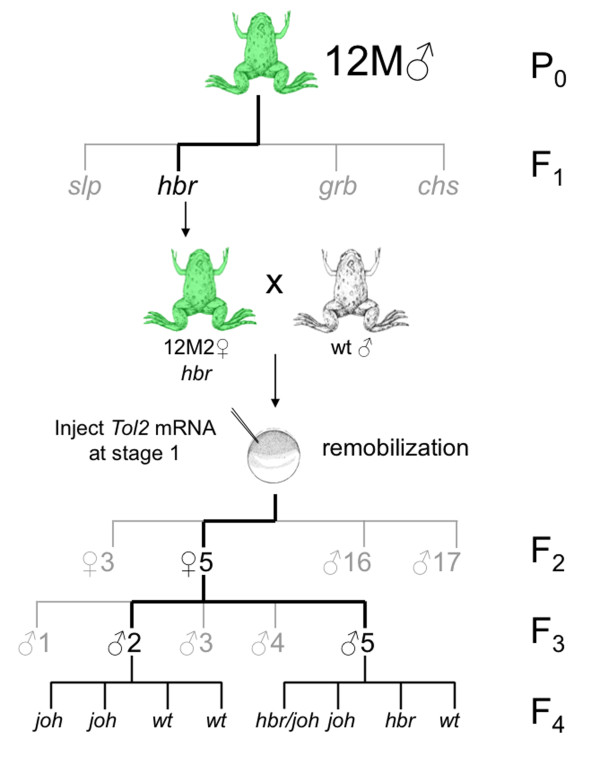
**Family tree of the 12M founder line with remobilization of progeny from F_1 _frog 12M2**. Schematic representation of the outcross and remobilization strategy used to characterize the 12M family. The founder (P_0_) 12M frog was outcrossed to yield the four independently-segregating alleles *slp*, *hbr*, *grb *and *chs*. An F_1 _frog that had a single *hbr *allele (12M2♀) was used to generate one-cell embryos for the *Tol2 *injection-mediated remobilization experiment. Outcross of remobilized F_2 _frog 12M2♀5 resulted in the generation of four new *Tol2*XIG transposon alleles. Serial outcross of the progeny of 12M2♀5 indicated that, in the absence of injected *Tol2 *transposase, the remobilized *Tol2 *transposons are stable in the *Xenopus tropicalis *genome and are transmitted to the progeny at the expected Mendelian frequencies.

Embryos from the outcross of 12M2♀5 were sorted based on their GFP expression pattern and tadpoles with the kidney pattern were raised to adults for subsequent outcross (family tree depicted in Figure 6). To assess whether the new remobilization events are stable in the genome and that, in the absence of *Tol2 *transposase activity, the novel integration events are inherited at the expected Mendelian frequencies, progeny from the outcross of two individual F_3 _males derived from 12M2♀5 (see Figure 6) were scored for GFP expression patterns and genomic DNA samples prepared from the F_4 _tadpoles were analyzed by PCR and Southern blotting. These analyses indicated that the remobilized alleles are inherited at the expected Mendelian frequencies and were stable in the genome (data not shown).

## Discussion

### *Tol2* transposons can be remobilized in *Xenopus tropicalis*

Our previous studies demonstrated that *Tol2 *is an efficient system for generating transgenic *Xenopus tropicalis *[33]. Here, we show that *Tol2 *transposons stably integrated into the frog genome are efficient substrates for remobilization following re-expression of the *Tol2 *transposase enzyme. Analysis of genomic DNA harvested from transgenic embryos injected at the one-cell stage with *Tol2 *transposase mRNA revealed that remobilization was very efficient. One hundred percent of injected transgenic animals showed novel re-integration events and we demonstrate that remobilization can be achieved in the progeny of transgenic male or female founders. Analysis of organs from an adult 'remobilized' frog indicated that the animals produced by the transposase mRNA injection technique are highly mosaic. The mosaic nature of the remobilization events is likely due to the stochastic activity of the *Tol2 *enzyme in the rapidly dividing *Xenopus *embryo. Messenger RNA encoding the transposase is injected at the one-cell stage and it is likely to take some time to accumulate sufficient *Tol2 *protein to catalyze the remobilization events. During this time, early cleavage stages may have occurred resulting in random remobilization events in different blastomeres of the developing embryo. The mosaic nature of the somatic remobilization events is reflected by the mosaic germline transmission of the re-integrated transposons. We demonstrated that the remobilized transposons can be passed through the germline of the injected transgenic frog and that these remobilized transposons are stable in the genome in the subsequent generations. The transmission of transposons through the germline of remobilized frogs frequently resulted in non-Mendelian inheritance of the GFP transgene in the resulting progeny (see table 1). Outcross of remobilized F_2 _animals was predicted to have resulted in the expected Mendelian segregation of the dominant GFP allele, that is 50% GFP-positive and 50% GFP-negative. Several of the outcrosses yielded transmission frequencies that were not significantly different from the expected rate of GFP-positive progeny (12M2♂1, 12M2♀2 and 12M2♀14). Genomic PCR analysis and Southern blot analysis indicated that only the parental (*hbr*) allele is present in the progeny of these animals indicating that remobilization has not occurred (data not shown). In other outcrosses the observed rate of transmission of the reporter to the progeny was significantly different from the expected rate (Chi-squared analysis p < 0.001 for lines 12M2♀5, 12M2♀6 and 12M2♂16, table 1). The variation in the expected rate of transgenesis may be due to the mosaic nature of the remobilization reaction following injection of the *Tol2 *transposase mRNA at the one-cell stage. For example, if the transposition reaction results in excision, but not re-integration, of the parental transposon in discrete blastomeres that will give rise to the germline, then a transmission rate lower than 50% may be anticipated. For example, progeny from founder line 12M2♀6 are 44.24% positive for GFP, suggesting a loss of GFP by excision but not reinsertion of the *Tol2 *transposon. It is also possible that remobilization has occurred in the germline of 12M2♀6 line but the novel insertion event has disrupted a genomic locus causing a loss of GFP positive embryos. Subsequent PCR and Southern blot analysis for the 12M2♀6 line, however, has failed to detect any evidence for transposon re-integration in this line (data not shown). Finally, a third class of outcross embryos was identified from the 'remobilized' 12M2 adults. Frogs 12M2♀5, 12M2♂16 and 12M2♂17 each produced GFP-positive tadpoles at a rate significantly higher than that expected for the outcross of a dominant heterozygous allele. Detailed analysis of the progeny from 12M2♀5 demonstrated that multiple remobilization events had occurred in the germline of this frog and were passed on to the offspring. To date, we have been unable to identify novel integration sites by PCR strategies and Southern blot analysis in the progeny from founder lines 12M2♂16 and 12M2♂17 (data not shown), even though population numbers suggest *Tol2 *remobilization is occurring in these lines.

The germline of remobilized F_2 _frog 12M2♀5 was highly mosaic and contained four novel integration events in addition to the parental (*hbr*) transposon. Approximately 22% of the GFP-positive progeny from this animal contained novel remobilization events. This indicates that the germline of this frog is mosaic for the transposon insertions, and as discussed above, likely resulted from random remobilization events occurring during early cleavage stages in the development of the 12M2♀5 tadpole.

The different inheritance frequencies of the remobilized alleles suggest that the remobilization events occurred at different times during the development of the transposase-injected embryo 12M2♀5. It is likely that the *joh *re-integration event occurred first as this is the remobilized allele most commonly seen in the outcross population. The *cen *allele is the next most commonly found allele and thus likely occurred after the *joh *remobilization event. The *fol *allele is commonly found with the *cen *allele indicating that the *fol *remobilization event most likely occurred in a blastomere derived from a prior *cen *remobilization event. As the *bru *allele is only seen either alone or with the parental *hbr *allele, this suggests that this remobilization event occurred in a blastomere that was not targeted by the other remobilization events (*joh*, *cen *and *fol*). This data clearly demonstrates that *Tol2 *transposons integrated into the *Xenopus tropicalis *genome are effective substrates for remobilization and the re-integration events are passed through the germline. Expression of the *Tol2 *transposase in early developing embryos results in random remobilization events and the germline of the resulting adult is mosaic for the parental and remobilized transposon transgenes.

**Figure 7:**
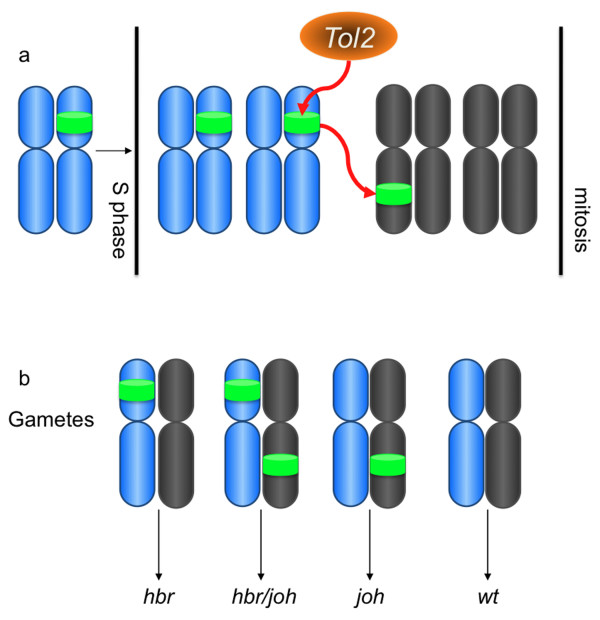
**Proposed mechanism for the increase in the number of transposons following injection-mediated *Tol2 *remobilization**. One-cell embryos harvested from an outcross of 12M2, with a single transposon integration (*hbr*), were injected with *Tol2 *mRNA. GFP-positive tadpoles were raised to adulthood and outcrossed to identify germline transmission of the remobilized transposon. Analysis of progeny from remobilized frog 12M2♀5 indicated that many of the offspring had inherited multiple copies of the *Tol2*XIG transposon. The *Tol2 *transposase uses a simple 'cut-and-paste' transposition mechanism and the apparent increase in transposon content in the progeny was unexpected. The likely explanation for the increase in number of *Tol2 *transposons is the remobilization of the substrate following DNA replication, but prior to mitosis (reviewed in [42]), in the blastomeres in the early embryo. DNA replication (S phase) results in two copies of the *Tol2 *substrate in each cell and one, or both, copies can be remobilized by the injected *Tol2 *transposase enzyme. During mitosis the transposon alleles are randomly segregated into the daughter cells. In the example depicted in the cartoon (a), the resident *Tol2 *transposon on the hypothetical blue chromosome is replicated during S phase and one copy is remobilized by the *Tol2 *transposase to a new location on a hypothetical grey chromosome. If the targeted blastomere gives rise to a germ cell, then the resulting gametes may contain different combinations of the transposon alleles (b) and the resulting tadpoles may have one or both of the integration events.

The most striking feature of the progeny that resulted from this outcross was the presence of multiple alleles of the *Tol2*XIG transposon in the same tadpole. The F_1 _frog that this line was derived from, 12M2, contained a single *Tol2 *transposon integration event, *hbr *(see Figure 4). How does a transposase that uses a simple 'cut-and-paste' transposition mechanism result in an increase in the number of transposon alleles in individual progeny? As with the mosaic nature of the remobilized frogs, the answer likely lies in the rapid development of the early *Xenopus *embryo and the propensity of *hAT *elements to mobilize during cell division [40-42]. The *Xenopus *embryo divides rapidly and synchronously for the first twelve cell cycles that are simple S (DNA synthesis) and M (mitosis) phases with no G (gap) phases. If *Tol2 *catalyzes remobilization of the resident *Tol2*XIG transposon in a blastomere after DNA synthesis but prior to mitosis, then, following random segregation of the sister chromatids, the resulting blastomere may inherit both the parental allele and the remobilized allele (Figure 7). Subsequent rounds of cell division and *Tol2 *enzymatic activity may further increase the complexity of the transposon content of an individual blastomere. If this blastomere gives rise to a germ cell, then the resulting gametes produced by this cell could contain multiple copies of the transposon transgene (Figure 7).

### Potential uses for *Tol2* remobilization strategies in the frog

Transposons offer efficient mechanisms for generating transgenic *Xenopus*. The established strategy for generating novel transgenic lines using transposon systems involves injected embryos at the one-cell stage with a cocktail containing a plasmid harbouring a transposon substrate and synthetic mRNA encoding the corresponding transposase enzyme. Micro-injection of plasmid DNA is toxic to the developing embryo and thus limits the amount of substrate that can be provided for the transposition reaction. Remobilization of transposons already integrated in the genome thus offers an attractive alternative to the co-injection approach. As illustrated in the examples shown here, the *Tol2*XIG transposon can act as an enhancer trap and remobilization of the transposon to novel loci can result in unique GFP expression profiles. Another potential benefit of the remobilization strategy is to take advantage of the 'local hopping' phenomenon to generate mutations in genes near the primary integration site. DNA 'cut-and-paste' transposons preferentially re-integrate at loci near the initial integration site. For example, remobilization of *Sleeping Beauty *transposons in the germline of mice results in approximately 50-80% of the re-integration events occurring within 5 cM either side of the primary integration site [52-55]. The 'local hopping' phenomenon has also been observed with *Tol2 *in several vertebrate species. In this study, we show several examples of re-integration of the *Tol2XIG *transposon to the same sequence scaffold as the initial transposition event in *Xenopus tropicalis*. 'Local hopping' of remobilized *Tol2 *transposons in the zebrafish germline has recently been reported by both the Kawakami and Korzh laboratories [35,56]. In mice, remobilization of resident *Tol2 *transposons in the germline frequently results in re-integration near the donor site [39].

Small-scale micro-injection based enhancer trap screens will be performed in the frog where novel patterns are scored in the progeny of remobilized animals and the flanking sequences are cloned to reveal the nearby genes. The *Soul Patch *(*slp*) transgenic line will be ideal for remobilization studies, as excision of the transposon from this locus and re-integration at a novel site will likely change the unique GFP expression pattern observed in this line [Additional file 3: Supplemental fig. S2]. The rate-limiting step in this approach is the micro-injection technique, and future efforts will be directed at the generation of transgenic frogs that express the *Tol2 *transposase in the *Xenopus *germline. The enzyme-expressing frogs can then be crossed with substrate transposon-harbouring frogs to generate double transgenic animals that have inherited both the enzyme and the substrate for remobilization. Outcross of these double transgenic frogs will result in progeny with novel expression patterns and remove the necessity for the micro-injection step. This double-transgenic (*Tol2 *substrate and *Tol2 *enzyme) remobilization approach has been used with success in both zebrafish and mice [35,39]. The advantage of pursuing this strategy in *Xenopus tropicalis *is the remarkable fecundity of this species, clutch sizes routinely surpass 2,000 embryos, and the longevity of the frogs, adult animals may live for twenty years or more in captivity. Combined with the exquisite embryological advantages of the *Xenopus *system, transposon-mediated gene and enhancer trap screening in the frog will provide an excellent combination of modern molecular genetics in a highly tractable developmental system.

## Conclusions

### *Tol2 *transposons stably integrated into the *Xenopus tropicalis *genome are substrates for remobilization

Transposon transgenesis techniques offer several advantages over other techniques for manipulating the frog genome. The 'cut-and-paste' mechanism of transposase enzymes such as *Tol2 *results in no plasmid vector sequences being integrated along with the transgene. The integrated transposons are also efficient substrates for excision and re-integration at novel sites. This has at least two important implications. First, as described above, the integrated transposon can be remobilized to perform gene and enhancer trap screens. Second, if integration of the transposon into a locus results in a mutant phenotype, the transposon can be remobilized from that site in order to rescue the phenotype and establish a direct link between the integration event and the mutation. In this manuscript, we demonstrate that *Tol2 *transposons integrated in the frog genome are effective substrates for remobilization.

### Remobilized *Tol2 *transposons are transmitted through the germline

Micro-injection of mRNA encoding the *Tol2 *transposase into one-cell *Tol2 *transgenic embryos results in the mosaic remobilization of the transposon from the parental locus. This is likely due to random remobilization in discrete blastomeres during early embryonic development. In order for these remobilization events to be passed through the germline, the enzymatic activity must occur in blastomeres fated to contribute to the germ cells. We demonstrated that remobilized transposons are indeed transmitted through the germline of remobilized *Tol2 *transgenic frogs that had been injected at the one-cell stage with *Tol2 *mRNA. The presence of multiple *Tol2 *transposons in individual progeny from a 'remobilized' frog, that was derived from a founder with a single integration event, indicates that the remobilization events have occurred in the developing blastomeres after S phase but prior to mitosis. The remobilization events are stable in the genome and can be segregated by serial outcross of the remobilized transgenic parent. The demonstration of efficient remobilization of *Tol2 *transposons in the *Xenopus tropicalis *genome is an important step in the development of transposon-based gene and enhancer trap screens in this tractable developmental model organism.

## Methods

### Husbandry and micro-injection of *Xenopus tropicalis*

*Xenopus tropicalis *tadpoles were maintained at 28°C in static tanks and were staged according to Nieuwkoop and Faber [57]. Adult animals were housed in a recirculating aquarium at 26°C. *In vitro *transcribed *Tol2 *transposase mRNA was prepared as previously described [33] using mMessage mMachine kits (Ambion) from a linearized pCS-TP plasmid [58]. Female *Xenopus tropicalis *animals were pre-primed with a 1:5 dilution of human chorionic gonadotropin (hCG) overnight and primed the day of injection with 200U of hCG. Fertilized eggs were obtained by natural outcross and microinjected with 500 pg of *Tol2 *mRNA at the one cell stage according to previous methods [43]. Injected eggs were allowed to heal at 28°C and transferred to tanks for growth at 28°C. The studies described in this manuscript were approved by the St. Jude Children's Research Hospital Animal Care and Use Committee.

### Southern blot hybridization

Genomic DNA was extracted from embryos by overnight digestion at 56°C with 0.5 mg/ml proteinase K, followed by phenol:chloroform extraction. Three micrograms of genomic DNA were digested overnight at 37°C with 15U of *Bgl*II or *Bam*HI (Promega) and separated on a 0.7% (w/v) agarose gel. Digested fragments were transferred overnight onto a Hybond N+ nylon membrane (GE Biosciences) with 20x SSC. Blots were probed with a ~700 bp GFP fragment labeled with ^32^P dCTP radionucleotide and the Megaprime labeling kit (GE Biosciences). After successive washes, blots were exposed onto a phosphoimager screen and processed on a Storm phosphoimager (Molecular Dynamics).

### Extension primer tag selection (EPTS) linker-mediated PCR

To determine the genomic integration site of transposons in *Xenopus tropicalis*, extension primer tag selection (EPTS) linker-mediated PCR was performed as described [43] with slight modifications. Genomic DNA samples were digested overnight with *Nla*III (New England Biolabs) or *Alu*I (New England Biolabs) and primer extension products were generated using the *Tol2 *biotinylated primer specific to the *Tol2 *5' arm sequence (5' BIO-AAA CTG GGC ATC AGC GCA ATT CAA T). One microliter (μl) of each primary PCR was used as templates for nested PCR reactions. Unique nested fragments were gel-purified, ligated to the pCR4-TOPO sequencing vector (Invitrogen) and miniprep DNA was extracted using the Promega Wizard miniprep kit. DNA sequencing was performed on all isolated DNA samples and aligned using Lasergene software. Identified sequences were queried against the JGI *Xenopus tropicalis *genome (ver 4.1; http://genome.jgi-psf.org/Xentr4/Xentr4.home.html) and scaffolds were assigned to chromosomes and linkage groups based on the genetic map developed at the University of Houston.

### Genomic PCR of identified integration sites

Primers were designed to genomic sequences adjacent to the integration site and within the transposon arms encompassing the identified EPTS LM-PCR fragment. Primers were designed to the predicted sequence of the 3' arm of the *Tol2 *transposon to further confirm the integration site. For the *Handlebar *(*hbr*) integration site, primers were designed to scaffold 98, (MJH1624, 5'-GCA CAG TCT GGG CTT TGT AT) and to the 3' transposon arm of *Tol2*, (MJH2153, 5'-AGT AGC GTG TAC TGG CAT TA) and annealed at 55°C. Primers were designed to the *Garibaldi *(*grb*) integration locus in scaffold 217 (217-2, 5'-TGT TGT TGT CGT CGT TCG TT) and within the 5' arm of the *Tol2 *transposon (217-1, 5'-CGC AAT TCA ATT GGT TTG GTA) and annealed at 52°C. For the *jovan heat *(*joh*) integration site located in scaffold 512, primers were designed to genomic sequence outside of the EPTS LM-PCR fragment (512f1, 5'-AAT AGT TTT CCC AGT AGC AAG TTA) and to the 5' *Tol2 *transposon arm (512tol2R1, 5'-CAA GGG AAA ATA GAA TGA AGT GAT) and annealed at 51°C. Primers were designed for the *centaure *(*cen*) integration site on scaffold 15, (15f2 5'-TCG ATG CAA ACT ATG TCA AAG AA) and to the 5' arm of *Tol2 *transposon, (t3r4, 5'-TAG CGT GTA CTG GCA TTA GAT T) and annealed at 52°C. Primers were designed to the *follique *(*fol*) integration site on scaffold 98, (n98f1, 5'-GCC CAT ATG CCT ACA GTG CCT CTA) and to the 5' arm of the *Tol2 *transposon, (t5-f6, 5'-AAA GTA AAA ATC CCC AAA AAT AAT), and annealed at 60°C. Approximately 200 ng of genomic DNA was used per PCR reaction. Conditions for genomic PCR for all primer sets were as follows: 95°C for 15 min., followed by 35 cycles of 94°C for 30 sec., annealing temperature indicated above for 30 sec., 72°C for 45 sec. Genomic DNA was amplified using the HotStart *Taq *polymerase (Qiagen) and resolved on agarose gels. All products were subcloned in pGEM-Teasy (Promega) and sequenced with M13F and M13R.

### Microscopy and Photography of *Xenopus tropicalis* embryos

Green fluorescent protein (GFP) expression was monitored using a Leica FLIII dissecting microscope. Images were obtained using a Nikon D5-5M color digital camera at the same aperture settings and exposure time in order to qualitatively assess variable GFP intensities.

## List of abbreviations

BB: basihyal-basibranchial cartilage; EPTS LM-PCR: Extension Primer Tag Selection Linker Mediated Polymerase Chain Reaction; GFP: Green Fluorescent Protein; hCG: human chorionic gonadotropin; OT: outflow tract; PBT: pharyngo-branchial tract; TSD: target site duplication.

## Authors' contributions

DAY carried out embryo injections, scored tadpoles, performed molecular analysis of transposon integration sites and helped prepare the manuscript. CMK performed molecular analyses of transposon integration sites, scored tadpoles and helped prepare the manuscript. EK performed embryo injections, scored progeny, assisted with molecular analyses and helped with general husbandry. HZ performed embryo injections and helped score progeny. AKS and DEW provided mapping data to assign sequence scaffolds to the *Xenopus tropicalis *linkage groups/chromosomes. PEM conceived the study, directed the project and wrote the manuscript. All authors read and approved the final manuscript.

## Supplementary Material

Additional file 1 **Supplemental Data**. Text file describing the four independently-segregating *Tol2*XIG integration events in founder 12M. Click here for file

Additional file 2 **Supplemental Figure S1 - The 12M founder has four independently-segregating *Tol2*XIG transposons each with a unique GFP expression pattern**. Outcross of the 12M founder resulted in the segregation of four independent *Tol2*XIG alleles and revealed unique GFP expression patterns associated with each integration event. (a) Schematic representation of the outcross of founder 12M to yield tadpoles with individual expression patterns. The individual patterns were named *Soul Patch *(*slp*), *Handlebar *(*hbr*), *Garibaldi *(*grb*) and *Chinstrap *(*chs*). Tadpoles were photographed at stage 51 and the figures are oriented with anterior towards the top of the panel. The intense GFP expression in the *slp *embryo in the basihyal basibranchial cartilage is labelled BB. The bright GFP expression in the leading edge of the *hbr *tadpole is indicated by the white arrow. The white arrowhead in the *chs *panel points to the GFP expression in the lower jaw of the tadpole. The eye is labelled in this panel to guide the reader. (b) Southern blot analysis of F_1 _tadpoles harbouring different combinations of the four transposons in founder 12M. Genomic DNA from individual tadpoles was digested with *Bgl*II and the resulting Southern blot was probed with a GFP probe. (c) EPTS LM-PCR was used to clone the genomic sequences flanking the transposon insertion sites in three of the four 12M alleles. The genomic DNA sequence flanking the transposon is indicated by the capitalized text and the sequence of the 5' end of *Tol2*XIG is shown in lowercase italics. Click here for file

Additional file 3 **Supplemental Figure S2 - GFP expression in the *Soul Patch *line**. GFP expression profile of the *Soul Patch *(*slp*) line derived from the *Tol2*XIG 12M founder. The EF-1α promoter in the *Tol2*XIG construct can be influenced by local regulatory elements near the transposon insertion site to override the normal ubiquitous expression of the GFP reporter. The *slp *allele has intense GFP expression in various cartilages in the developing tadpole (Stage 51 shown). (a) *slp *results in intense GFP expression in the provisionally identified basihyal basibranchial (BB) cartilage at the midline of the head. (b) Schematic representation of the tadpole head skeleton indicating the relative position of the basihyal basibranchial cartilage (adapted from Weisz, 1945 [59]). The *slp *allele also results in intense GFP expression in the cartilage supporting the tentacle (c and e) and the cartilage supporting the gill arches (d, white arrows). GFP expression is clearly visible in the outflow tract (OT) of the heart in *slp *tadpoles (f). Images a, c, d and f were taken on a fluorescent dissecting microscope and e is an overlay of a confocal image with the corresponding bright-field view. Click here for file

Additional file 4 **The *jovan heat *(*joh*) allele has a *Tol2*XIG transposon integrated near the HNF1β gene **(a) Schematic representation of the *Tol2*XIG integration event in *joh *(not to scale). The transposition reaction resulted in integration of the transposon in intron 9 of a novel HEAT motif-containing gene on scaffold 512:565147. *In situ *hybridization with antisense RNA probes generated to the HEAT repeat cDNA indicated low-level ubiquitous expression of the HEAT motif-containing gene that lacked robust expression in the developing kidney (data not shown). The HNF1β gene flanks the 3' end of the HEAT motif-containing gene and is approximately 46 kb from the *Tol2*XIG transposon. (b) *In situ *hybridization for HNF1β expression during *Xenopus *development shows intense staining in the developing kidney [47]. Antisense RNA probes were synthesized from a *Xenopus laevis *HNF1β cDNA (IMAGE 4959359). *In situ *stained albino *Xenopus laevis *embryos shown at stages 27 and 35 (anterior is facing to the right, dorsal up). Arrows point to the developing kidney. Click here for file
