# Supplementary material for: Remobilization of Tol2 transposons in Xenopus tropicalis
Source: BMC Dev Biol. 2010 Jan 22;10:11. doi: 10.1186/1471-213X-10-11 (PMC2848417; doi:10.1186/1471-213X-10-11)
Supplement: Additional file 1 — Supplemental Data. Text file describing the four independently-segregating Tol2XIG integration events in founder 12M. [file 1471-213X-10-11-S1.doc]

# Supplemental data

*Founder 12M has four independently-segregating Tol2XIG transposons*

Sequential outcross of the 12M line resulted in segregation of these alleles to reveal four distinct GFP expression patterns corresponding to four unique integration events in founder 12M [Additional file 2: Supplemental fig. S1]. The first pattern identified from founder 12M was named *Soul Patch* (*slp*) as the intense expression of GFP in a cartilage within the head, provisionally identified as the basihyal-basibranchial (BB) cartilage [Additional file 2: Supplemental figs. S1a (left panel) and Additional file 3: Supplemental fig. S2a, b [1]], resembled the curious facial hair style worn below the lower lip. The *Soul Patch* integration produces intense GFP expression in various neural crest-derived tissues throughout the body [Additional file 3: Supplemental fig. S2]. These include the cartilages supporting the tentacles [Additional file 3: Supplemental fig. S2c, e] and gill arches [Additional file 3: Supplemental fig. S2d]. The *Soul Patch* allele also results in intense GFP expression in the outflow tract (OT) of the heart [Additional file 3: Supplemental fig. S2f], another neural crest-derived tissue. Three additional GFP expression patterns associated with unique integration events were isolated from founder 12M. In keeping with the facial hair theme inspired by *Soul Patch* (*slp*), these were named *Handlebar* (*hbr*), *Garibaldi* (*grb*) and *Chinstrap* (*chs*) [Additional file 2: Supplemental fig. S1a]. *Handlebar* has widespread reporter expression with very intense GFP expression in the leading (anterior) edge of the pharyngo-branchial tract (PBT). The *Garibaldi* allele results in intense ubiquitous GFP expression. The GFP expression profile of *Chinstrap* is predominantly restricted to the lower jaw.

Southern blot analysis of F_1_ embryos selected by visual GFP inspection from an outcross of the 12M founder identified individual or combinations of the independently-segregating transposons [Additional file 2: Supplemental fig. S1b]. Extension Primer Tag Selection Linker Mediated PCR (EPTS LM-PCR) was used to identify the integration site for each of the observed expression patterns [Additional file 2: Supplemental fig. S1c] [2]. Three of the patterns (*hbr, slp,* *grb)* have flanking sequences consistent with canonical integration of the *Tol2XIG* transposon into the *Xenopus tropicalis* genome. The *hbr* locus is located at the end of scaffold 98 (base pair position 2851245) approximately 60 kb from a gene with similarity to the human pleckstrin homology domain containing family G member 1 gene.

Integration site analysis for *slp* revealed transpsoson integration within a repeat found throughout the *Xenopus tropicalis* genome. BLASTN results of linker-mediated PCR sequences with matches greater than 99% identity revealed three potential integration sites with a best-match of 99.18% (1087 bp identity over 1096 bp) to scaffold 42 at base pair position 284862; within an intergenic region near the MCLF2/OstII gene. The two other potential integration sites for the *slp* locus shared greater than 99% identity to the 1096 bp of cloned flanking sequence (scaffold 78:1241804 (1086/1096 = 99.09%) and scaffold 14:92499 (1086/1096 = 99.09%)). Long-range PCR amplification strategies were attempted to distinguish between the three potential repeat regions identified in the BLASTN searches of the *Xenopus tropicalis* genome, however these approaches were unsuccessful and the identity of the *slp* integration site remains unknown.

EPTS LM-PCR fragments for the *Garibaldi* (*grb*) allele identified the integration site in the 3’ untranslated region of the SMAD7 gene (scaffold 217:1488798), a member of the inhibitory SMAD family of signalling molecules. Scaffold 217 maps to linkage group 1 (chromosome 1) of the *Xenopus tropicalis* genome.

The *Chinstrap (chs)* allele contains a linear *Tol2*XIG fragment with flanking plasmid sequences and likely represents a random integration event that is not a product of a true transposition reaction (data not shown). Due to presence of flanking plasmid sequence, we were unable to identify genomic locus of the *chs* integration.

# References

1. PB Weisz: **The development and morphology of the larva of the South African clawed toad, Xenopus laevis. I. The third-form tadpole**. *Journal of Morphology* 1945, **77**:163-192.

2. DA Yergeau, E Kuliyev, PE Mead: **Injection-mediated transposon transgenesis in Xenopus tropicalis and the identification of integration sites by modified extension primer tag selection (EPTS) linker-mediated PCR**. *Nat Protoc* 2007, **2**:2975-86.
