# Supplementary figures and images for: Remobilization of Tol2 transposons in Xenopus tropicalis
Source: BMC Dev Biol. 2010 Jan 22;10:11. doi: 10.1186/1471-213X-10-11 (PMC2848417; doi:10.1186/1471-213X-10-11)

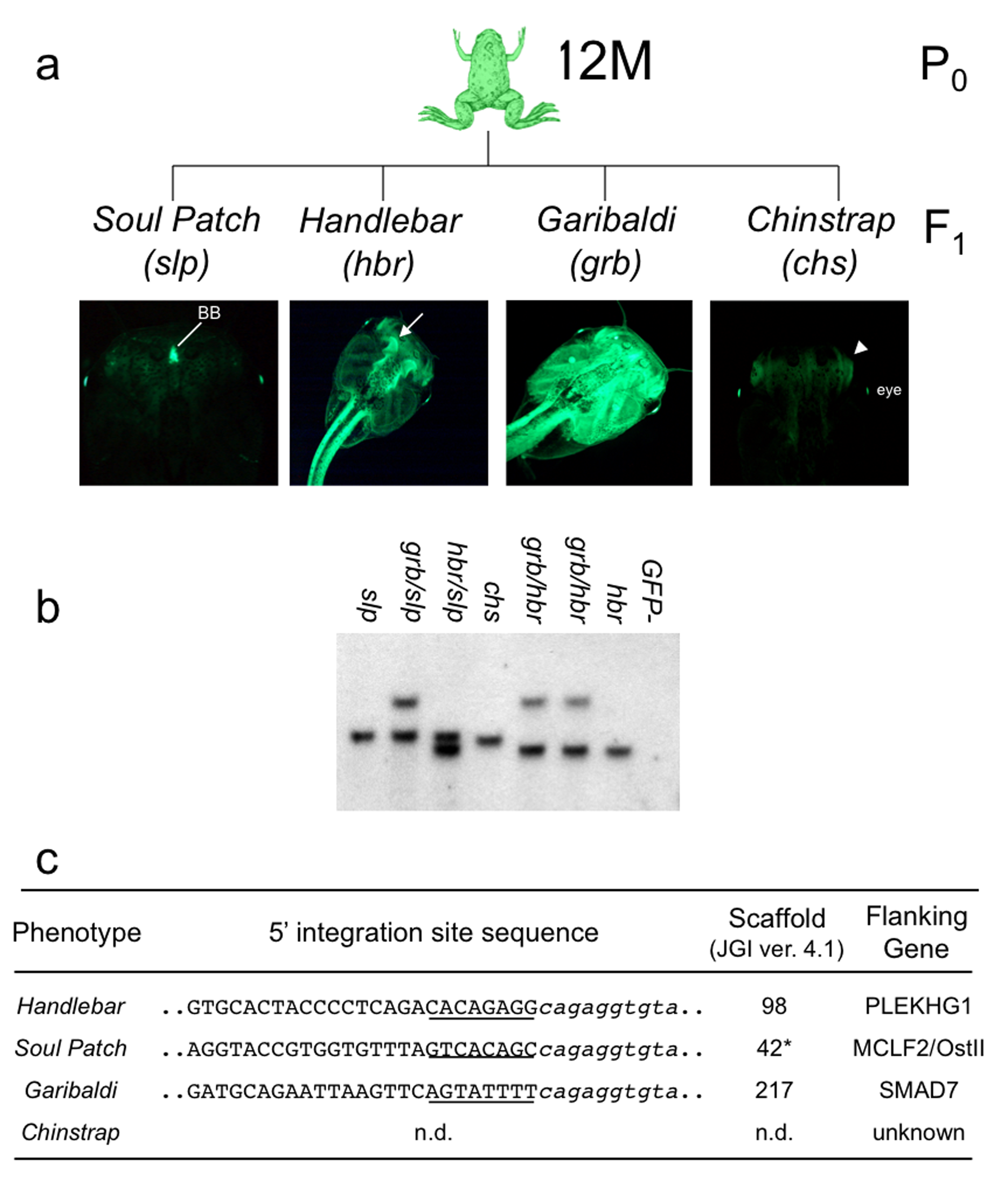

Supplement: Additional file 2 — Supplemental Figure S1 - The 12M founder has four independently-segregating Tol2XIG transposons each with a unique GFP expression pattern. Outcross of the 12M founder resulted in the segregation of four independent Tol2XIG alleles and revealed unique GFP expression patterns associated with each integration event. (a) Schematic representation of the outcross of founder 12M to yield tadpoles with individual expression patterns. The individual patterns were named Soul Patch (slp), Handlebar (hbr), Garibaldi (grb) and Chinstrap (chs). Tadpoles were photographed at stage 51 and the figures are oriented with anterior towards the top of the panel. The intense GFP expression in the slp embryo in the basihyal basibranchial cartilage is labelled BB. The bright GFP expression in the leading edge of the hbr tadpole is indicated by the white arrow. The white arrowhead in the chs panel points to the GFP expression in the lower jaw of the tadpole. The eye is labelled in this panel to guide the reader. (b) Southern blot analysis of F1 tadpoles harbouring different combinations of the four transposons in founder 12M. Genomic DNA from individual tadpoles was digested with BglII and the resulting Southern blot was probed with a GFP probe. (c) EPTS LM-PCR was used to clone the genomic sequences flanking the transposon insertion sites in three of the four 12M alleles. The genomic DNA sequence flanking the transposon is indicated by the capitalized text and the sequence of the 5' end of Tol2XIG is shown in lowercase italics. [file 1471-213X-10-11-S2.tiff]

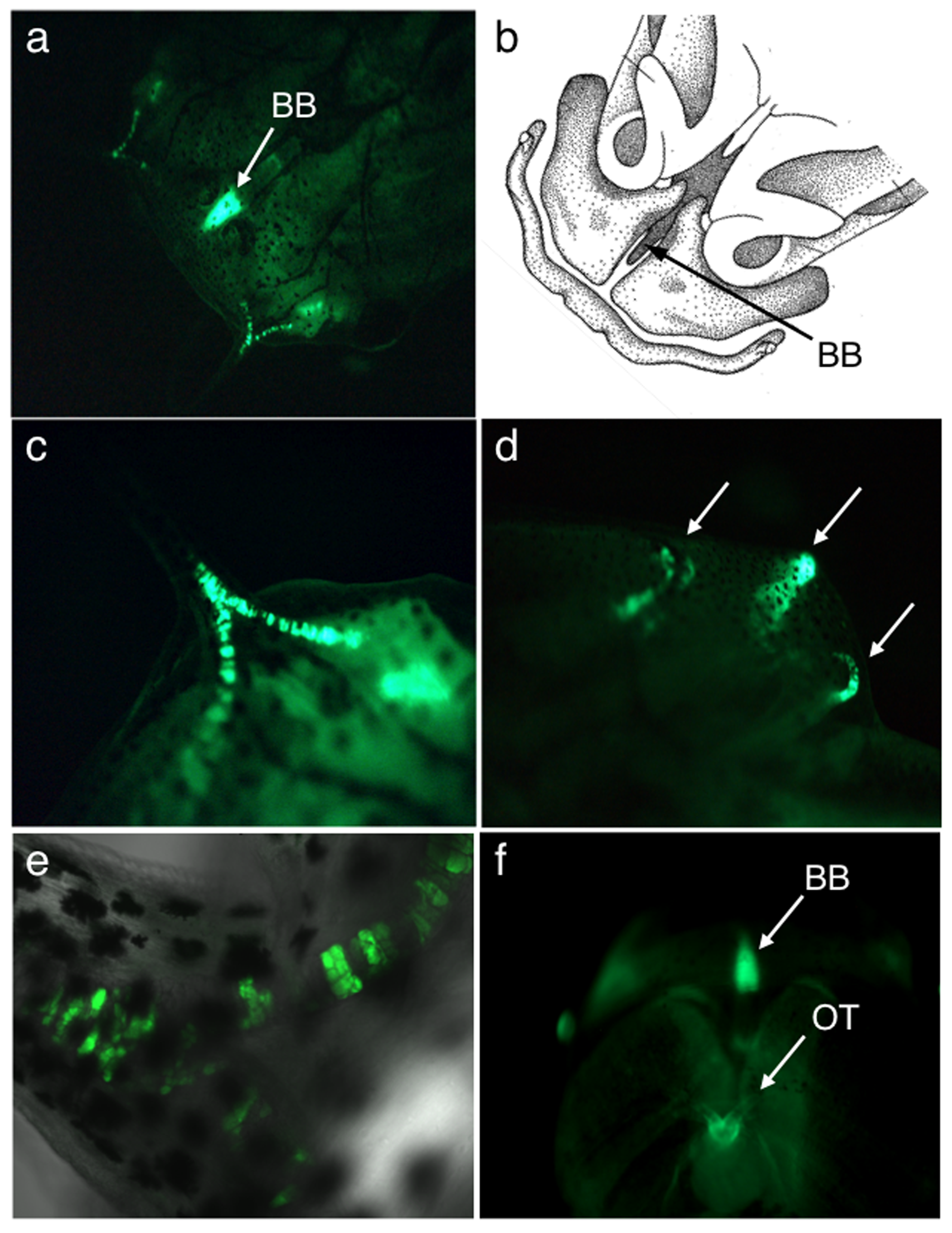

Supplement: Additional file 3 — Supplemental Figure S2 - GFP expression in the Soul Patch line. GFP expression profile of the Soul Patch (slp) line derived from the Tol2XIG 12M founder. The EF-1α promoter in the Tol2XIG construct can be influenced by local regulatory elements near the transposon insertion site to override the normal ubiquitous expression of the GFP reporter. The slp allele has intense GFP expression in various cartilages in the developing tadpole (Stage 51 shown). (a) slp results in intense GFP expression in the provisionally identified basihyal basibranchial (BB) cartilage at the midline of the head. (b) Schematic representation of the tadpole head skeleton indicating the relative position of the basihyal basibranchial cartilage (adapted from Weisz, 1945 [59]). The slp allele also results in intense GFP expression in the cartilage supporting the tentacle (c and e) and the cartilage supporting the gill arches (d, white arrows). GFP expression is clearly visible in the outflow tract (OT) of the heart in slp tadpoles (f). Images a, c, d and f were taken on a fluorescent dissecting microscope and e is an overlay of a confocal image with the corresponding bright-field view. [file 1471-213X-10-11-S3.tiff]

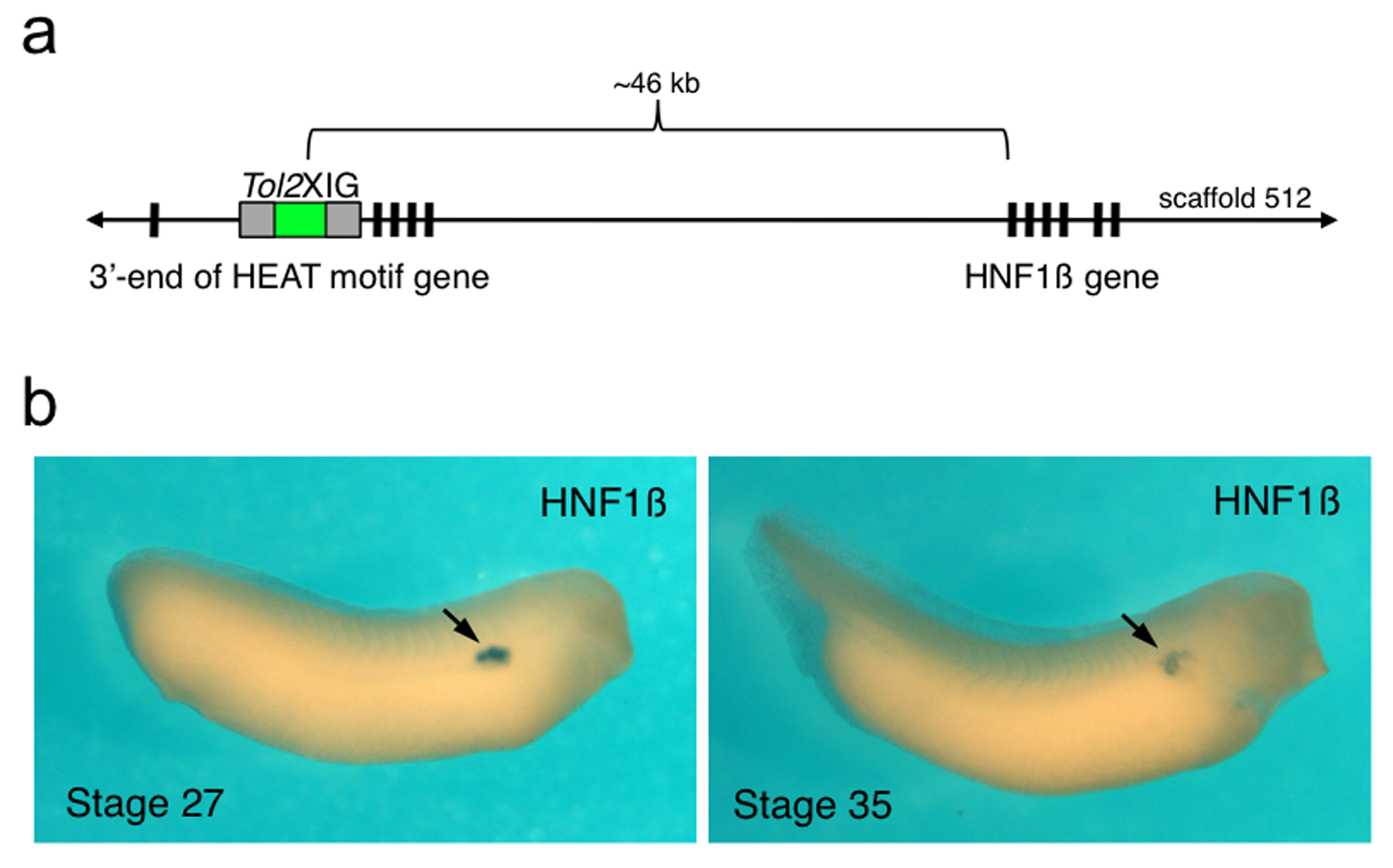

Supplement: Additional file 4 — The jovan heat (joh) allele has a Tol2XIG transposon integrated near the HNF1β gene (a) Schematic representation of the Tol2XIG integration event in joh (not to scale). The transposition reaction resulted in integration of the transposon in intron 9 of a novel HEAT motif-containing gene on scaffold 512:565147. In situ hybridization with antisense RNA probes generated to the HEAT repeat cDNA indicated low-level ubiquitous expression of the HEAT motif-containing gene that lacked robust expression in the developing kidney (data not shown). The HNF1β gene flanks the 3' end of the HEAT motif-containing gene and is approximately 46 kb from the Tol2XIG transposon. (b) In situ hybridization for HNF1β expression during Xenopus development shows intense staining in the developing kidney [47]. Antisense RNA probes were synthesized from a Xenopus laevis HNF1β cDNA (IMAGE 4959359). In situ stained albino Xenopus laevis embryos shown at stages 27 and 35 (anterior is facing to the right, dorsal up). Arrows point to the developing kidney. [file 1471-213X-10-11-S4.tiff]
